# Harnessing technology to enhance oral health literacy among Afghan women: an interprofessional community-engaged initiative

**DOI:** 10.3389/fpubh.2025.1594767

**Published:** 2025-08-27

**Authors:** Moshtagh R. Farokhi, Andrew E. Muck, Hamsini Nathan, James Yan, Alvin Estacio, Ruoxuan Su, Melanie Stone, Nandini Mandlik, Heidi Worabo

**Affiliations:** ^1^Department of Comprehensive Dentistry, San Antonio Refugee Health Clinic, School of Dentistry, University of Texas Health Science Center at San Antonio, San Antonio, TX, United States; ^2^Department of Emergency Medicine, University of Virginia, Charlottesville, VA, United States; ^3^School of Dentistry, University of Texas Health Science Center at San Antonio, San Antonio, TX, United States; ^4^Joe R. & Teresa Lozano Long School of Medicine, University of Texas Health Science Center at San Antonio, San Antonio, TX, United States; ^5^South Central AHEC (Area Health Education Center), University of Texas Health Science Center at San Antonio, San Antonio, TX, United States; ^6^Department of Family & Community Medicine, Joe R. & Teresa Lozano Long School of Medicine, University of Texas Health Science Center at San Antonio, San Antonio, TX, United States; ^7^Department of Pediatrics, Joe R. & Teresa Lozano Long School of Medicine, University of Texas Health Science Center at San Antonio, San Antonio, TX, United States; ^8^Office for Faculty Excellence, School of Nursing, University of Texas Health Science Center at San Antonio, San Antonio, TX, United States

**Keywords:** cultural practices, health communication, refugee health, health literacy, stakeholder participation

## Abstract

**Introduction:**

Ongoing socio-political instability has resulted in a growing influx of Afghan women with limited literacy skills resettling in the United States (U.S.). These women face considerable barriers in accessing oral and general healthcare services, exacerbated by limited literacy. This study aimed to explore Afghan women’s challenges in accessing healthcare services and develop a technology-enabled intervention to enhance their health.

**Methods:**

An interprofessional (IP) team of researchers collaborated with community advocates and leaders to gather participants’ insights about preventive health. Participants identified *WhatsApp* as their preferred platform for receiving health information. Trained interpreters were employed to bridge cultural and linguistic gaps. The first phase involved conducting pre-intervention surveys to assess participants’ experiences with the availability, accessibility, and affordability of healthcare services. Culturally tailored oral health messages were developed using the interactive *Canva* platform. These videos incorporated messages in English, Pashto, and Farsi that were shared through *WhatsApp*, to accommodate varying literacy levels. The second phase emphasized hands-on demonstrations to illustrate oral hygiene techniques, and a customized rubric was used to assess participants’ competency. Pre- and post-surveys for the pilot study assessed changes in knowledge and behavior, which helped refine the intervention protocol. Follow-up interviews were conducted six months post-intervention, assessing variations in outcome.

**Results:**

Forty-three Afghan women aged 19 to 57 participated in the study. Demographic data revealed that 58% identified Pashto as their primary language, 46% had received little to no formal education, and 77% were homemakers. Key barriers to healthcare access included financial constraints (91%), limited English proficiency (70%), low literacy levels (63%), and transportation challenges (56.3%). Post-intervention data revealed statistically significant improvements in participants’ oral hygiene and dietary knowledge (*p* < 0.05). Assessment of brushing and flossing techniques indicated increased performance scores from pre-to post-intervention (*p* < 0.001). At the six-month post-intervention follow-up, 20 women participated. 80% (*n* = 16) reported reduced consumption of sugary beverages, while 95% (*n* = 19) indicated improved oral hygiene practices.

**Conclusion:**

This study supports the effectiveness of a culturally responsive, technology-facilitated oral health intervention in enhancing self-care behaviors among Afghan refugee women. The findings suggest that healthcare providers should adopt patient-centered, community-engaged approaches to advance health outcomes. Technology-based interventions can effectively address constraints in health literacy.

## 1 Introduction

Refugees resettling in a new country often encounter numerous stressors, including conflicting cultural norms, unfamiliar religious customs, and disrupted social support systems. These stressors significantly impact their overall health and wellbeing, including their oral health, as they navigate complex healthcare systems while facing financial, linguistic, and systemic barriers (1). In the United States (U.S.), despite existing resettlement support services (2), refugees face environmental challenges such as unfamiliar healthcare practices, dietary adjustments, sociocultural misunderstandings, and limited health literacy. These factors hinder self-care, restrict access to dental services, and contribute to inadequate oral hygiene practices (3, 4).

Such barriers are particularly relevant for the over 100,000 Afghans who have resettled in the U.S., many of whom have endured significant physical and emotional trauma during their displacement (5, 6). Between 2010 and 2022, the Afghan immigrant population in the U.S. nearly quadrupled, from approximately 54,000 to 195,000. In 2021 alone, 76,000 evacuated Afghans received humanitarian parole status to enter the United States (6).

Afghan cultural norms are deeply rooted in religious practices, which in turn shape daily oral hygiene practices (7). One such normalized practice is using chewing sticks as a traditional toothbrushing technique. The sticks are chewed on one end to create a frayed, brush-like tip used to clean teeth, while flossing is less familiar (7, 8).

Offering culturally sensitive oral health education and information about the U.S. healthcare system requires a comprehensive approach that addresses transportation, financial, and language barriers (9). Healthcare providers face unique challenges when treating patients from other cultures (10). Prioritizing oral health as a core, integral component of wellbeing and actively empowering marginalized groups, especially women, directly aligns with the United Nations’ Sustainable Development Goals of SDG 3 (good health and wellbeing), 5 (equality), and 10 (reduced inequalities) (11).

While previous studies have examined oral health disparities among refugees (12), few have implemented or evaluated culturally tailored and technology-driven interventions designed explicitly for Afghan women with limited literacy and English proficiency.

Emerging evidence supports the use of informed health advisors to enhance oral health knowledge and attitudes among Culturally and Linguistically Diverse (CLD) communities, effectively bridging communication gaps (13). However, educational outreach to Afghan women is hindered by low literacy levels and limited formal education (14, 15).

Health literacy refers to an individual’s ability to access, understand, evaluate, and apply health information to make informed decisions about their health (16). Strong health literacy skills are closely linked to better health outcomes, while limited health literacy is associated with poor health outcomes, higher healthcare costs, and greater health disparities (17). Similarly, digital literacy is the ability to locate, assess, and effectively communicate information using digital technologies (18). Emerging research highlights the critical role that digital communication tools play in enhancing health literacy, ultimately empowering individuals to take a more active and informed role in managing their health (19).

Digital communication tools, such as *WhatsApp Messenger*, present promising opportunities for delivering health outreach by facilitating rapid, multimodal communication through text messages, images, and video calls. Such platforms can enhance patients’ comprehension of general and oral health concepts; hence, improving clinical outcomes (20, 21). Technology-based interventions such as *YouTube* and other social media platforms have proven effective in delivering health education, raising awareness, and encouraging positive health behavior change (22, 23). This study sought to identify the factors that enhance Afghan women’s access to oral healthcare services and to promote improvements in their oral health literacy and practices.

## 2 Materials and methods

### 2.1 Intervention design and development

This quasi-experimental intervention utilized digital technology platforms and community engagement to address oral health disparities among Afghan women. Digital content was crafted with a focus on preventive empowerment self-care (21–23), emphasizing three critical components: comprehensibility (cognitive), manageability (behavioral), and meaningfulness (motivational). These pillars align with the framework proposed by Eriksson et al. (24), ensuring that the intervention addresses the multidimensional process of health knowledge acquisition and behavior change.

**Figure 1:**
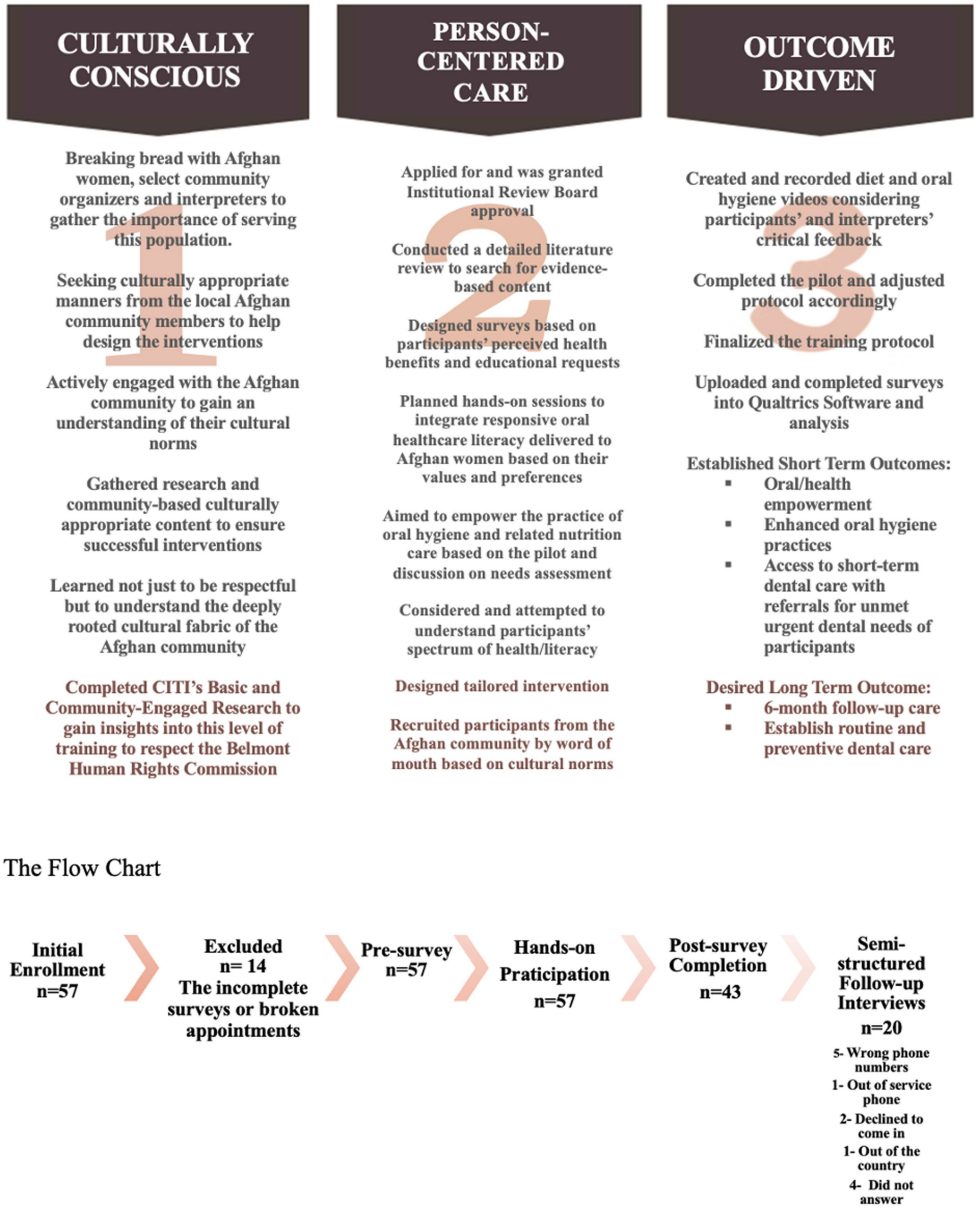
The planning process and flow chart.

Motivational interviewing style interventions were developed in collaboration with the local Afghan community leaders. Afghan women guided the research questions and evaluation metrics during “breaking bread” lunches that promoted relationship-building and trust (Figure 1). Community events, including English as a Second Language (ESL) classes and oral health roundtables, provided opportunities for socialization and needs assessment. The study was pilot tested at such gatherings to refine the research design and uncover potential problems with the intervention protocol, the study procedure, or data collection through participant feedback.

Community events, including English as a Second Language (ESL) classes and oral health roundtables, provided opportunities for socialization and needs assessment. To minimize interpretation confusion, a simplified Likert scale with two response options (agree/disagree) addressed literacy and education limitations.

### 2.2 Participant recruitment and interventional design

Participants were recruited at our Refugee Health Clinic (Clinic, 9), which has provided services in the local community for over a decade. Inclusion criteria were adult Afghan women. This interventional study enrolled a convenience sample of 43 Afghan women (Figure 1) seeking urgent oral healthcare at the Clinic. During the study phase, 346 Afghan resettled women attended the Clinic, and 57 elected to participate.

Each participant received one-on-one training focused on oral hygiene instructions, and nutrition. IP providers applied a kinesthetic learning approach using tooth models or typodonts to demonstrate proper oral hygiene instructions. They also played two *Canva* videos in English, Pashto, and Farsi with narrations in Pashto and Farsi to reinforce the connections between general and oral health and diet as related to toothbrushing and adequate flossing.

- Video 1 demonstrated proper brushing and flossing techniques.
- Video 2 covered nutritional health: food group portion sizes, fresh versus processed foods, meal timing, and diet tips for cavity prevention.

The videos were used as training tools during the session and disseminated as *WhatsApp* links for take-away messages.

Using a rubric-based evaluation, calibrated providers, including the dental faculty, dental students, and medical students, assessed participants’ tactical oral hygiene skills immediately after the hands-on training (Appendix A) (25). The 3-point scale measured the amount of toothpaste, brush bristle stiffness, brushing time, and technique, coverage of tooth surfaces, and flossing techniques. The author developed Rubric Form (Appendix A), assessed, evaluated, and tracked participants’ progress with their oral hygiene skills.

### 2.3 Measurement and instruments

Calibrated interpreters facilitated the administration of the surveys for participants with limited English proficiency and literacy limitations.

#### 2.3.1 Pre-intervention measures

**Figure 2:**
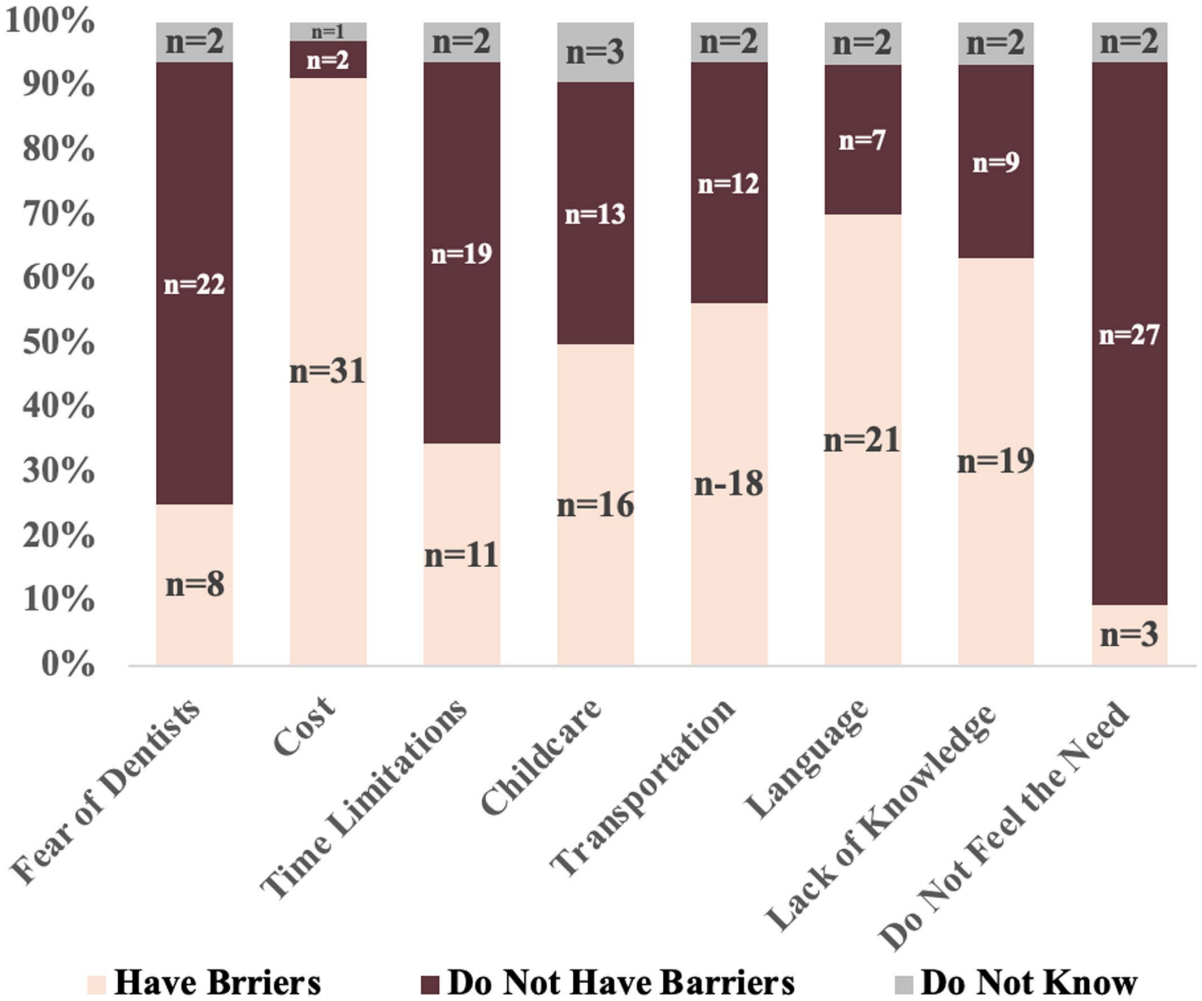
Study participant barriers to healthcare (*n* = 32).

After providing informed consent, the participants completed pre-intervention surveys, including validated tools (26–28) to assess the quality of life, oral health perceptions, and access to care. The World Health Organization (WHO) WHOQOL-BREF measured the domains such as physical health, environmental context, and perceived wellbeing (26). Survey questions, co-developed with interpreters and community organizers, also evaluated culturally informed practices and baseline knowledge of oral and nutritional health (Tables 1, 2) (29–31). The pre-survey, consisting of 27 questions, focused on sociodemographics, education, and employment status (Table 1), access to oral healthcare (4), oral health and hygiene (10), nutritional health (11, Table 2), healthcare availability (5), accessibility (6), and affordability (2, Table 3). It also examined participants’ barriers to both oral and general health (3), Figure 2).

**Table 1:** Participants’ pre-intervention, sociodemographics score percentages and numbers.

Demographics of Afghan women participants	*N* (%)
Age	*N* = 43
18–29	*N* = 16, 37%
30–49	*N* = 24, 56%
50–64	*N* = 3, 7%
Afghan Languages Spoken	*N* = 43
Pashto	*N* = 25/43, 58%
Dari	*N* = 17/43, 39%
Farsi	*N* = 1/43, 3%
Education	*N* = 43
No Formal or Informal education	*N* = 20/43, 46%
Completed primary school (grades 1–6)	*N* = 8/43, 19%
Completed upper secondary school (grades 10–12)	*N* = 8/43, 19%
Some level of college education	*N* = 7/43, 16%
English as a Second Language (ESL) Class Training	
Had at least one opportunity and availability to study (ESL)	*N* = 20/43, 46.5%
Could not attend any ESL classes	*N* = 23/43, 53.5%
Current in training	
Full ESL training	*N* = 5/43, 12%
High school General Educational Development programs	*N* = 1/43, 2%
Employment	*N* = 43
Not employed and not looking for work or stay at home	*N* = 33/43, 77%
Not employed, but looking for work	*N* = 7/43, 16%
Employed currently-only in the Shoe Factory completing stickwork	*N* = 3/43, 7%
US residency status	*N* = 43
Less than one year	*N* = 16/43, 37%
1–2 years	*N* = 23/43, 55%
2–5 years	*N* = 3/43, 8%
Access to the training sessions-transportation	*N* = 43
Drove their vehicles	*N* = 4/43, 10%
Received transportation from their husbands	*N* = 15/43, 35%
Carpooled with a friend or neighbor	*N* = 11/43, 25%
Used public transport-buses	*N* = 3/43, 7%
Walked to the training session	*N* = 10/43, 23%
Access to oral healthcare	*N* = 43
Dental care service access	
Attended the dental Clinic-within walking distance	*N* = 22/43, 51%
Received urgent care from private practices	*N* = 13/43, 30%
Did not visit a dentist at all	*N* = 8/43, 19%
Dental health-seeking behavior	
Visited a dentist when they experienced pain	*N* = 20/43, 46.5%
Did not visit a dentist due to financial limitations	*N* = 23/43, 53.5%
Dental Provider preference	
Agreed to be treated by a male dentist in the absence of a female dentist	*N* = 22/43, 51%
Refused to be treated by a male dentist	*N* = 8/43, 19%
Had no preference to the gender of the provider	*N* = 13/43, 30%
Oral hygiene knowledge	*N* = 41
Flossing	
Had knowledge about flossing but did not know how to floss	*N* = 7/41, 17%
Believed they knew how to floss but not certain	*N* = 30/41, 73%
Had never heard of flossing	*N* = 4/41, 10%
Primary purpose for toothbrushing	
Have a cleaner mouth and brighter smile	*N* = 8/41, 19.5%
Not sure why to brush	*N* = 33/41, 80.5%

**Table 2:** Proportion of correct responses for the oral and health related assessment of knowledge among participants in the Pilot (*n* = 11) and Study (*n* = 32) groups Pre and Post the intervention.

	Pilot group		Study group	
	Pre	Post	Pre	Post
	Proportion of correct responses (%)			
Oral hygiene				
**Understanding that bleeding gums is oral infection**	**27.3**	**27.3**	**39.3**	**60.7**
Daily toothbrushing habits				
Purpose of toothbrushing	45.5	27.3	39.3	67.9
Frequency of toothbrushing	72.7	81.8	57.1	78.6
Duration of toothbrushing	45.5	81.8	25.0	57.1
Most important time of the day to brush teeth	81.8	100.0	67.9	75.0
Amount of toothpaste to use	63.6	100.0	39.3	53.6
Miswak Sticks as brushing substitute	27.3	63.6	25.0	32.1
Flossing Technique	36.4	72.7	28.6	64.3
**Best time to floss each day**	**0.0**	**27.3**	**0.0**	**46.4**
**Frequency of professionally cleaned teeth**	**72.7**	**100.0**	**57.1**	**78.6**
DIET				
Cariogenic beverages and foods promoting cavities				
Sugary foods and cavities	54.5	90.9	85.7	92.9
Drinking acidic beverages that promote cavities	45.5	45.5	67.9	53.6
Protective factor choices to reduce cavities				
Drinking cariostatic beverages that halt or reduce cavities	54.5	63.6	21.4	42.9
Snacks to keep teeth and mouth healthy	72.7	72.7	82.1	92.9
Dairy products for healthy teeth and gingivae or gums	90.9	100.0	82.1	92.9
**Benefits of community water fluoridation**	**45.5**	**36.4**	**7.1**	**50.0**
**Importance of eating fruits and vegetables on oral/health**	**–**	**–**	**35.7**	**64.3**
**Fruit juices consumption-cariogenic or cavity promoting**	**–**	**–**	**25.0**	**39.3**
**Effects of high sugar consumption on oral/health**	**–**	**–**	**64.3**	**78.6**
**Effects of processed foods on oral/health**	**–**	**–**	**46.4**	**64.3**
**GERD oral/health side effects-eating close to bedtime**	**–**	**–**	**46.4**	**82.1**

The values shown in bold within this table indicate the highest levels of increased effect observed.

**Table 3:** Proportion of responses to the quality of life assessment questions by Pilot group (*n* = 11) and Study group (*n* = 32).

Quality of life assessment	Pilot group	Study group	Overall
	Proportion (%)	Proportion (%)	Proportion (%)
Availability of care			
Ability to access adequate dental care for self and family members.	45.5	89.3	76.9
Ability to access adequate medical care for self and family members.	18.2	89.3	69.2
Knows where to seek dental care.	36.4	85.7	71.8
Has knowledge of where to seek medical care.	27.3	89.3	71.8
Is satisfied with their ability to find one good dentist, physician or doctor to treat the whole family.	18.2	50.0	41.0
Accessibility of care			
Knows where to seek care for a dental emergency.	27.3	82.1	66.7
Knows where to seek care for a medical emergency.	27.3	85.7	69.2
Lives in proximity to dentists’ offices.	18.2	82.1	64.1
Lives in proximity to physicians’ offices.	27.3	85.7	69.2
Has difficulty traveling to the dentist’s office.	36.4	67.9	59.0
Has difficulty traveling to the physician’s office.	36.4	67.9	59.0
Affordability of care			
Can afford adequate dental care.	9.1	14.3	12.8
Can afford adequate medical care.	9.1	21.4	17.9

#### 2.3.2 Post-intervention measures

The post-intervention survey, excluding sociodemographic questions (Tables 1, 2), was administered immediately after the intervention training. The post-survey assessed changes in knowledge and behavior using the same simplified Likert format.

Six months post-intervention, semi-structured interviews were conducted for 20 of the participants. Interviews explored participants’ (1) the capacity of the intervention outcomes, (2) the relative benefit/s of the intervention (28), and (3) the ability to be self-reliant (32).

### 2.4 Data management and analysis

The IP providers and interpreters recorded survey responses directly into a password-protected Qualtrics system. Responses were de-identified to ensure confidentiality. Descriptive statistics were used to summarize (1) quality of life, (2) oral health knowledge, and (3) healthcare access barriers. The pre-and post-interventional surveys were administered digitally using password-protected Qualtrics links. Wilcoxon matched-pairs signed-rank tests compared pre-and post-survey results. Analyses were performed using Stata 18.0 (StataCorp LLC, College Station, TX). Informed consent was obtained verbally and in writing at each session, as IRB approval of HSC-20230224NRR.

## 3 Results

### 3.1 Access to oral healthcare services

#### 3.1.1 Participant demographics and barriers to accessing oral healthcare

The sociodemographic information of the participants is reported in Table 1. A total of 43 Afghan women participated in the intervention. Generally, participants were Afghan women ranging in age from 19–57 years old who mostly spoke Pashto (58%), presented with established U.S. residency status of 1–2 years (55%), had limited formal education (46%), and were not employed and not looking for work (77%). Few participants (23%) reported working outside the home as teachers, school principals, physicians, medical assistants, midwives, or tailors when they lived in Afghanistan. Transportation to training sessions emerged as a significant barrier, with participants relying on husbands or friends for carpooling.

Participants’ barriers to accessing oral healthcare (Table 1; Figure 2) ranked by the participants included cost (91%), limited English proficiency (70%), low oral health literacy (63%), transportation challenges (56.3%), and childcare responsibilities (50%).

#### 3.1.2 Dental care utilization and treatment-seeking behavior

Access, as the lack of dental care, was a primary study objective. The majority (51%) of participants attended the Clinic’s urgent dental care services because of of walking or availability to access, 46.5% visited a dentist only when they experienced pain in an emergency, and the majority (70%) preferred female dental providers (Table 1).

The in-depth interviews revealed that the participants’ husbands play a significant role in their healthcare experiences, highlighting their influence on decisions and support system. Additionally, the study uncovered critical insights into the healthcare behaviors of Afghan refugee women, whose oral hygiene practices were often deprioritized due to overwhelming household responsibilities, particularly extensive childcare duties.

#### 3.1.3 Healthcare availability, accessibility, and affordability trends

The availability, accessibility, and affordability of healthcare evaluations based on the Quality of Life Assessment are reported in Table 3. Overall, participants reported moderate availability of resources such as personnel and technology to address their oral health needs, and moderate accessibility in receiving timely and appropriate healthcare services to achieve optimal health outcomes. However, only 41% were satisfied with their ability to find a single provider to treat the whole family, and 59% reported having difficulty traveling to both a dentist’s and a physician’s office. The participant level of affordability of healthcare services was also low, with 12.8% reporting they could not afford dental care and 17.9% reporting they could not afford medical care. Comparing dental and medical groups, the Study group reported more availability and access to dental and medical care. However, dental and medical affordability of healthcare services was low for both the Pilot and Study groups (dental: 9.1 and 14.3%, respectively; medical: 9.1 and 21.4%, respectively).

### 3.2 Oral health literacy

#### 3.2.1 Oral hygiene practices and knowledge gained

Participant knowledge improved notably from pre-to post-survey in several areas, including (1) understanding of gingivitis, proper brushing and flossing techniques, and the benefits of fluoride, and (2) recognition of Miswak stick usage as a traditional oral hygiene tool.

A tremendous increase in knowledge was in identifying the best time to floss daily, rising from 0 to 46% in the Study group to 0 to 3% in the Pilot group (Table 2).

Oral hygiene practices and diet knowledge were assessed using a survey administered before and after the intervention and scored based on the number of correct responses (Table 2). In both the Pilot and Study groups, increases in the proportion of correct responses were observed for several knowledge questions. For the Pilot group, the largest increases in the proportion of correct responses were seen in the amount of toothpaste to use (63.6 to 100%), sugary foods as cavity promoting agents (54.5 to 90.9%), the recommended duration of toothbrushing (45.5 to 81.8%), and the benefits of flossing teeth (36.4 to 72.7%). The data indicates a notable decline in knowledge scores about health-related practices, specifically toothbrushing, which decreased from 45.5 to 27.3%. Additionally, awareness of the benefits of community water fluoridation also experienced a reduction, dropping from 45.5 to 36.4%. These findings highlight the difficulties related to effectively communicating intricate health information. For the Study group, the highest increases in the proportion of correct responses were observed for daily flossing (0 to 46.4%), benefits of community water fluoridation (7.1 to 50.0%), and flossing technique (28.6 to 64.3%). Pre-intervention surveys revealed that 80.5% of participants were unaware of the primary purpose of toothbrushing (Table 2).

#### 3.2.2 Dietary knowledge

For the Study group, the highest increases in the proportion of correct responses about diet were observed for the Gastroesophageal Reflux Disease (GERD) condition (GERD) and oral/health side effects from eating close to bedtime (46.4 to 82.1%). The knowledge score decreased for the question about drinking acidic beverages and cavities, likely due to participants’ positive perceptions of drinking juices’ health benefits. Post-intervention dietary knowledge significantly improved as participants’ understanding of the importance of fruit and vegetable intake increased from 9.4 to 43.8%, a 34.4% increase. The Knowledge about fluoride benefits improved from 18.8 to 50%, a 32% increase. The median knowledge scores increased significantly for the Study group (9 to 14) and the Pilot group (9 to 11).

Both pre-and post-surveys were scored for each participant to evaluate the intervention’s effectiveness. A Wilcoxon matched-pairs signed-rank statistical test was performed and reported in Table 4. For the Pilot group, a significant increase in median oral hygiene knowledge and overall knowledge was observed (*p* = 0.01 and *p* < 0.01, respectively). For the Study group, median knowledge scores for oral hygiene (*p* < 0.01), diet (*p* < 0.01), and overall (*p* < 0.01) significantly increased after the intervention.

**Table 4:** Comparison of pre-and post-intervention median knowledge assessment scores among participants in the Pilot (*n* = 11) and Study (*n* = 32) groups.

Category	Pilot group			Study group		
	Pre	Post	*p*-value	Pre	Post	*p*-value
	Median score (IQR)			Median score (IQR)		
Oral hygiene	4.0 (3.0)	7.0 (1.0)	0.01*	3.5 (2.0)	6.0 (2.0)	<0.01*
Diet	4.0 (2.0)	4.0 (2.0)	0.39	6.0 (3.5)	8.0 (3.5)	<0.01*
Overall	9.0 (6.0)	11.0 (3.0)	<0.01*	9.0 (4.0)	14.0 (6.0)	<0.01*

Median survey scores were calculated for each category by summing the number of correct responses for each corresponding question. Exact *p*-values were calculated using the Wilcoxon matched-pairs sign-rank statistical test. IQR = Interquartile Range – explains the spread of the middle half of the distribution. *denotes a statistically significant *p*-value for *p* < 0.05.

### 3.3 Six month follow-up to assess oral health behavior

Of the 43 participants, 20 completed the follow-up interviews six months post-intervention. Of the 20, 80% (*n* = 16) reported occasional sugary drink consumption, while 20% (*n* = 4) reported none, 75% (*n* = 15) consumed sugary foods occasionally, and 25% (*n* = 5) abstained entirely. 85% (*n* = 17) brushed at least once daily, and 40% (*n* = 8) flossed at least once daily. 80% (*n* = 16) used fluoridated toothpaste once or more per day, and 65% (*n* = 13) had visited a dentist since the intervention; 35% (*n* = 7) had not. During the follow-up process, participants identified their remaining dental needs, which included pain (*n* = 4), restorations or fillings (*n* = 5), and professional tooth cleanings (*n* = 3).

## 4 Discussion

This community-engaged, person-centered intervention illuminated the barriers Afghan refugee women face in accessing and utilizing oral and general healthcare services in the United States. The key challenges identified include conflicting cultural norms, transportation barriers, limited proficiency in the dominant language, low health literacy, and financial constraints, which impacted the participants’ ability to access and understand healthcare services (9, 33–35).

### 4.1 Cultural norms and language access

This study highlighted that, despite participants’ high perceived access to oral health care, many encountered financial barriers that impeded their ability to afford such care. These findings imply that the cost of dental services plays a critical role in shaping participants’ health-related behaviors and care-seeking patterns among individuals. Limited language proficiency emerged as a significant factor impacting participants’ integration and perception of overall wellbeing. Participants faced challenges navigating the U.S. healthcare system due to unfamiliarity with its structure, services, and expectations (Table 3) (36). These barriers frequently led to delayed treatment-seeking, with care sought only when the pain became intolerable—a trend consistent with oral health behavior observed in other refugee communities (5, 10).

### 4.2 Person-centered and culturally responsive strategies

The novelty of this interventional study was its intentional alignment with participants’ religious, cultural, and logistical realities. Given most participants’ substantial homemaking responsibilities, training and interviewing sessions were deliberately scheduled outside prayer and meal preparation periods. Mothers were permitted to bring children to training sessions, reducing participants’ childcare barriers, as providers helped connect participants to local clinics and establish a “dental or medical home,” for them.”

Childcare burdens significantly impacted participants’ ability to attend appointments and complete surveys, echoing a common theme across low-income and refugee populations (37, 38). This insight reinforces the necessity of integrating childcare support and flexible scheduling into health interventions and the interconnectedness of factors that impact vulnerable populations. Participants also reported relying on informal networks of friends, neighbors, or family for childcare services during health visits (38). The main reason 23 women could not complete their longitudinal six-month follow-up care was childcare restrictions.

### 4.3 Family dynamics and decision-making

Afghan men traditionally hold financial and logistical responsibilities for their families and are often consulted regarding healthcare decisions. While this dynamic may initially appear at odds with Western ideals of autonomy, many participants described their husbands as supportive allies rather than authoritative decision-makers. This notion emphasizes that providers should avoid cultural assumptions and recognize context-specific gender dynamics (39, 40) when treating such population. For these reasons, the videos included English text so the husbands and English-speaking participants could be fully informed.

Moreover, significant literacy and language gaps amplified participants’ reliance on their husbands’ opinions. Hence, traditional gender dynamics should be considered, particularly how these roles could inhibit access to care. Providers adapted by ensuring that the content delivery was interpreter-assisted, delivered in Pashto and Farsi, and accessible via the *WhatsApp* platform suggested by the participants.

### 4.4 Oral health promotion and practice model

This study considered the traditional Afghan oral hygiene habits with Islamic practices like mouth rinsing during ablution, while incorporating science-backed preventive measures of fluoride toothpaste. Educational materials provided instruction on brushing, flossing, diet, and fluoride use, aligning with WHO recommendations of Basic Package of Oral Care, which promotes Affordable Fluoride Toothpaste (AFT), and indicating it’s significant reduction in the incidence of dental caries and periodontal disease (41).

### 4.5 Empowerment through interpretation

A notable success of this study was its use of trained community organizers and interpreters, many of whom had prior experience serving as cultural mediators in Afghanistan while working with the U.S. Troops. Their contributions were invaluable in both content delivery and trust-building. By incorporating participant voices into the message design, the intervention reduced cultural miscommunication and enhanced the relevance and uptake of oral health messages (42).

## 5 Implications

The structural burdens contributed to poor self-care routines and underutilization of preventive dental services. One of the most significant findings was the participants’ strong preference for receiving health education content via *WhatsApp* to be viewed again within the comfort of their home environments.

The successful use of multimedia content delivered through mobile platforms highlights the need for healthcare providers to invest in accessible and culturally appropriate educational tools. Such tools reflect the social contexts and support oral and nutritional health promotion beyond clinical settings that bridge the digital divide and circumvent the social isolation often experienced by refugee women while fostering engagement and autonomy.

Through relationship-building efforts with interpreters, community organizers, and participants, the providers created efforts extending well beyond health education, such as trust, empowerment, and long-term health advocacy.

Aligned with the World Health Organization’s Basic Package of Oral Care and the United Nations Sustainable Development Goals as SDGs 3, 5, and 10 (11), this interventional study design contributed to a broader global agenda of improving and promoting health. Notably, during the urgent care dental exam and triage phase, dental conditions observed by the provider dentist included fractured teeth, the reported frequent consumption of cariogenic foods, and lack of preventive care. Psychosocial stressors, often overlooked, emerged as hidden but powerful drivers of health behaviors. Such stressors, compounded by emotional and environmental distress, can lead individuals to feel a loss of self-identity and disconnection from traditional values (43).

The model of care used in this study highlights the value of community engagement as a mechanism for sustainable change. Technology becomes more than a communication tool; it becomes a dignified communication bridge to health promotion, integration, and content sharing. Despite challenges related to literacy and access to preventive care, routine follow-up metrics, such as health outcomes, could offer a more comprehensive evaluation.

Initially focused on increasing oral healthcare and preventive care outreach, the IP team recognized a deeper opportunity to transform this intervention into an empowerment strategy. As the process evolved, the strategy shifted to a community-engaged advocacy approach, with tools, knowledge, and a platform to reclaim participant agency over their oral and overall health.

## 6 Lessons learned and limitations

This intervention offered valuable insights into Afghan refugee women’s oral health needs and lived experiences by utilizing motivational interviewing and patient-provider cultural humility encounters while promoting oral and general health. Language and literacy barriers were addressed through tailored communication tools or visual aids. The intervention also enhanced the providers’ depth of cultural humility training and engagement.

A key lesson learned was the critical role of creative, culturally appropriate resources in helping refugee women adapt to unfamiliar healthcare systems and social environments. Many participants reported feeling isolated and overwhelmed when navigating complex health systems while managing extensive domestic and childcare responsibilities. These experiences are consistent with broader refugee literature that identifies structural exclusion and social disconnection as significant challenges for resettled populations (44, 45).

The lack of childcare support during interviews frequently led to overcrowded and occasionally chaotic environments, which could impact participants’ ability to focus. However, this challenge also produced unexpected positive outcomes: older children who observed the interviews became informal learners, absorbing health information alongside their mothers. This intergenerational exposure represents a subtle, indirect benefit, signaling the potential for family-centered health promotion approaches.

While the findings identify barriers for this population in a specific U.S. resettlement context, their applicability to other refugee communities or geographic areas should be cautiously approached.

Despite the relatively small size (*n* = 43) and the application of a convenience sample, depth of engagement and a comprehensive approach enhanced the outcome impact—one-on-one interviews allowed for a personalized and respectful data collection process. Hence, reliance on scripted and calibrated interpreters was the only alternative, it may present as response bias. While the study focuses on Afghan refugee women, it may not fully account for the experiences of other refugee groups facing similar barriers. Nonetheless, it could be adapted for populations with literacy challenges. Finally, to achieve equitable and effective implementation, careful consideration of accessibility, digital literacy, and data privacy is essential.

## 7 Conclusion

This community-driven interventional study highlighted the powerful intersection of grassroots outreach and digital engagement in reaching underserved populations. By leveraging existing networks, partnering with local organizations, and utilizing accessible platforms such as *WhatsApp*, the initiative effectively disseminated multimedia health content. This model of care outlined the impact of collective community-engaged action in promoting health and improving the wellbeing of resettled communities; however, ongoing education and support mechanisms are essential for long-term success.

This humanistic and upstream interventional model of care not only met immediate health needs but also initiated critical, broader conversations about integrating oral and general health services within vulnerable communities. The interventional study presented a scalable model suitable for displaced or linguistically challenged populations.; however, addressing the language, childcare, and cultural barriers encountered before implementation is critical.

Technology platforms, such as digital health tools and virtual consultation deliverables for refugees and other marginalized populations, can overcome structural barriers to healthcare, leading to improved oral and general health outcomes and potentially addressing language barriers, and geographical limitations (46).

Ultimately, by fostering a multi-agency interprofessional collaboration for this (46) interventional study this interventional study reinforces that empowering refugee women through education, engagement, and person-centered strategies can serve as a blueprint for responsive, inclusive healthcare that honors the dignity and resilience of displaced communities.

## Data Availability

The original contributions presented in the study are included in the article/supplementary material, further inquiries can be directed to the corresponding author.
